# Zero and Minimal Fluoroscopic Approaches During Ablation of Supraventricular Tachycardias: A Systematic Review and Meta-Analysis

**DOI:** 10.3389/fcvm.2022.856145

**Published:** 2022-04-11

**Authors:** Dorottya Debreceni, Kristof Janosi, Mate Vamos, Andras Komocsi, Tamas Simor, Peter Kupo

**Affiliations:** ^1^Heart Institute, Medical School, University of Pécs, Pécs, Hungary; ^2^Cardiac Electrophysiology Division, Department of Internal Medicine, University of Szeged, Szeged, Hungary

**Keywords:** meta-analysis, paroxysmal supraventricular tachycardia, catheter ablation, zero fluoroscopy, zero fluoroscopy ablation

## Abstract

**Introduction:**

Catheter ablations for cardiac arrhythmias are conventionally performed under fluoroscopic guidance. To guide these procedures, zero/minimal fluoroscopy (Z/MF) approaches have become available, using three-dimensional electroanatomical mapping systems. Our aim was to conduct a meta-analysis comparing these two different methods for the treatment of paroxysmal supraventricular tachycardia (SVT).

**Methods:**

Electronic databases were searched and systematically reviewed for studies comparing procedural parameters and outcomes of conventional, fluoroscopy-guided vs. Z/MF approaches in patients undergoing electrophysiology (EP) procedures for SVTs. The random-effects model was used to derive mean difference (MD) and risk ratios (RRs) with 95% confidence interval (CI).

**Results:**

Twenty-four studies involving 9,074 patients met our inclusion criteria. There was no difference between the groups in terms of acute success rate (RR = 1.00, 95% CI, 0.99–1.01; *p* = 0.97) and long-term success rate (RR: 1.01, 95% CI, 1.00–1.03; *p* = 0.13). Compared to the conventional method, zero-and-minimal fluoroscopy (Z/MF) ablation significantly reduced fluoroscopic time [MD: −1.58 min (95% CI, −2.21 to −0.96 min; *p* < 0.01)] and ablation time [MD: −25.23 s (95% CI: −42.04 to −8.43 s; *p* < 0.01)]. No difference could be detected between the two groups in terms of the procedure time [MD: 3.06 min (95% CI: −0.97 to 7.08; *p* = 0.14)] and the number of ablation applications [MD: 0.13 (95% CI: −0.86 to 1.11; *p* = 0.80)]. The complication rate was 1.59% in the entire study population and did not differ among the groups (RR: 0.68, 95% CI: 0.45–1.05; *p* = 0.08).

**Conclusions:**

The Z/MF approach for the catheter ablation of SVTs is a feasible method that reduces radiation exposure and ablation time without compromising the acute and long-term success or complication rates.

## Introduction

Catheter ablation has evolved as the standard treatment method for paroxysmal supraventricular tachycardia (SVT) owing to its low complications and high success rate (1). These procedures are conventionally performed using a fluoroscopy-guided approach, exposing both patients and medical staff with a potentially dangerous amount of ionizing radiation. Prolonged exposure to radiation may increase the chance of dermatitis, cataracts, and congenital defects, and. it can increase the risk of cancer in the exposed individuals (2).

Although this risk can be reduced by applying various forms of radiation protection described by the as low as reasonably achievable (ALARA) principle (3), based on recent publications, radiation protection is still not optimal in cardiac electrophysiology (EP) (4).

A notable development can be observed in terms of the three-dimensional (3D) electroanatomical mapping (EAM) systems of the past decade. The EAM systems can significantly reduce radiation dose and fluoroscopy time during procedures, and early studies showed that the zero-and-minimal fluoroscopy (Z/MF) approach during EP procedures is a safe and effective method. A previous meta-analysis including 2,261 patients from 10 trials published in 2016 showed reduced fluoroscopic and ablation time using Z/MF ablation for the treatment of cardiac arrhythmias, whereas there was no difference in procedure time and acute and long-term success rates compared to conventional, fluoroscopy-guided ablation procedures (5). Following this meta-analysis, further important studies–including prospective, randomized, and multicenter trials–have been published by comparing these two different strategies.

To gain further insight into the low fluoroscopy approach to catheter ablation, we aimed to study the subgroup of patients with supraventricular arrhythmias and we conducted a meta-analysis to compare the safety, efficacy, and procedural parameters between patients with SVT who underwent catheter ablation procedures either with Z/MF or with fluoroscopy guidance.

## Methods

### Search Strategy

Electronic databases [PubMed, Excerpta Medica Database (EMBASE), Cochrane Central Register of Controlled Trials (CENTRAL)] were searched for relevant articles between January of 2000 and July of 2021. The search string was “zero-fluoroscopy or near-zero fluoroscopy or fluoroless or non-fluoroscopic” and “electrophysiology or electrophysiological” and “catheter ablation” and “or supraventricular or supraventricular tachycardia or paroxysmal supraventricular tachycardia”. We extended the search with the reference list of the relevant studies. Duplicates and review publications were excluded. We performed the analyses according to the Preferred Reporting Items for Systematic Review and Meta-Analyses (PRISMA) guidelines (6).

In this meta-analysis, we included studies that accomplished the following criteria: (1) patients who underwent EP study and/or catheter ablation for paroxysmal SVT, atrioventricular nodal reentrant tachycardia (AVNRT), atrioventricular reentrant tachycardia (AVRT), atrial tachycardia (AT), or cavotricuspid isthmus-dependent atrial flutter (AFL); (2) patients having at least 1 Z/MF -only and one conventional fluoroscopy-only arm; (3) randomized or non-randomized prospective studies and retrospective studies enrolling consecutive patients; and (4) studies written in English. Case reports, letters, abstracts, conference presentations, and ablation of atrial fibrillation or left atrial macroreentrant tachycardia were excluded.

Zero fluoroscopy was defined as no radiation and was used during the procedure. Under “minimal fluoroscopy”, we meant those cases in which, although the operator planned to follow zero-fluoroscopy strategy, the limited use of radiation became necessary during the procedure.

### Data Acquisition and Statistical Analysis

Study selections and data acquisition were performed independently by two reviewers (D.D. and P.K.). Disagreements were resolved by consensus.

### Endpoints of Interest

The primary outcome of the study was the acute success rate. Secondary outcomes included procedural parameters: “skin-to-skin procedure time” (minutes); “ablation time”, that is the sum of ablation time during the entire procedure (seconds); “application number”, which means the sum of the radiofrequency delivery; and “total fluoroscopy time” (minutes), “fluoroscopy dose” (mGy), and “fluoroscopy exposure” [dose area product (DAP), cGy/cm^2^]. Complications and long-term success rate were also analyzed.

### Statistical Analysis

We performed the analyses according to the PRISMA guidelines using the dmetar 0.0.9, meta 4.15-1, and metaphor 2.4-0 packages with R statistical software 4.0.3 (6, 7). Pooled treatment effects as mean difference (MD) on continuous data and risk ratios (RRs) for binary end-points were compared with their 95% confidence intervals (CIs). The significance of the pooled estimates was determined by the Z-test, and *p* < 0.05 was considered as statistically significant. We quantified the possibility of heterogeneity between studies and the proportion of inter-study variability by Cochran's Q-statistic and *I*^2^ statistics, respectively. The latter describes the percentage of total variation across studies due to heterogeneity rather than due to chance. Values of *I*^2^ < 25% were considered as low and values of *I*^2^ > 75% were considered as high. The choice of the random-effects model was made based on the consideration that the true effect of low-dose fluoroscopy strategy may vary from study to study influenced by heterogeneity of the included trials. The random-effects model provides more conservative and robust results and accounts better for inter-study differences, however, it also tends to have a higher impact of small study bias. Thus, statistical inference was based on the results of random-effects model analyses. However, we also present results of fixed-effect modeling as a sensitivity exercise. In the random-effects models, the DerSimonian–Laird tau 2 estimator was used to estimate the variance of the distribution of true effect sizes and account for inter-study variability. To assess the stability of acquired effect estimates, a leave-one-out sensitivity analysis was applied. Quality assessment was performed with Cochrane's tool for assessing bias, wherein studies are scored as high, low, or unclear risk of bias in five domains: selection, performance, detection, attrition, and reporting. Funnel plot was drawn to assess publication bias, and asymmetry was assessed by visual estimation and by Egger's linear regression test. In case of any of these suggested substantial asymmetry, Duval and Tweedie's trim-and-fill procedure was applied. With imputing missing studies into the funnel plot until symmetry is reached again, this helps to estimate what the actual effect size would be had the “missing” small studies been published.

## Results

### Study Characteristics

**Figure 1:**
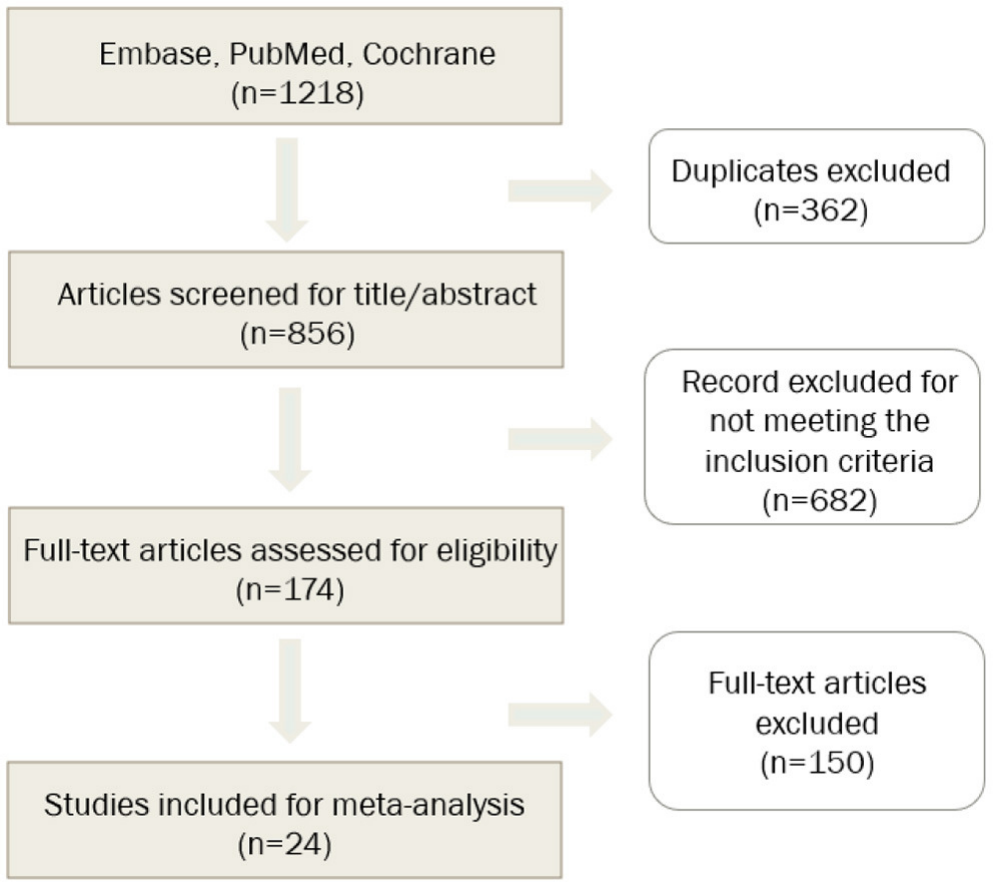
Flowchart of study selection.

Twenty-four studies involving 9,074 patients were analyzed (Figure 1) (8–31). Among the 24 included studies, three were randomized controlled studies, whereas the rest were observational trials. The main characteristics of the trials are summarized in Table 1. The EnSite NavX system was used in 18, the EnSite Precision System in 3, the EnSite Velocity System in 3, the CARTO System in 8, the Rhythmia System in 1, and the MediGuide System in one studies. The mean length of the follow-up period varied between 42 and 1,584 days.

**Table 1:** Study and patients' characteristics of the included trials.

**References**	**Design**	**Patient number**	**Number of EAM systems**	**Procedure type**	**Operators**	**Operators' experience with EAM systems**	**EAM system**	**Use of ICE**	**Sex (male/female number)**	**Median follow-up (days)**	**Mean age**	**Mean BMI or weight**	**ZF success**
Earley et al. (8)	Single center, prospective randomized	96	45	AVNRT, AVRT, AFL, Other	2	NA	EnSite NavX	NA	53/43	42	52 ± 15; 47 ± 16	NA	100%
Smith and Clark (10)	Single center, retrospective, non-randomized	60	30	AVNRT, WPW, concealed pathway	NA	NA	EnSite NavX	NA	25/35	90	12.6 ± 4.35	21.4; 18.4 (BMI)	80%
Álvarez et al. (9)	Single center, prospective, non-randomized	100	50	AVNRT	NA	NA	EnSite NavX	NA	20/80	180	59.15 ± 15	NA	98%
Kwong et al. (11)	Single center, retrospective, non-randomized	388	318	AVRT, AVNRT	NA	NA	EnSite NavX	NA	219/167	NA	11.9 ± 4.2 12.2 ± 3.7	47 ± 19.6 53.1 ± 22.4 (kg)	NA
Stec et al. (12)	Multicenter, prospective, non-randomized	902	188	AVNRT, WPW/AVRT, AFL, AT	NA	NO	EnSite NavX	NO	413/489	240 ± 156; 330 ± 171	45 ± 21; 52 ± 18	NA	95%
Casella et al. (13)	Multicenter, prospective, randomized	262	134	AVNRT, Right AP, Left AP, AFL, AT	NA	YES	EnSite NavX	NA	110/152	360 ± 132	36.3 ± 10.4 35.4 ± 10.4	24.4+ 4.4 23.5+ 4.4 (BMI)	72%
Schoene et al. (14)	Single center, prospective, randomized	40	20	AFL	2	YES	MediGuide	NA	34/6	180	65.2 ± 12	28.8 ± 4 (BMI)	NA
Romero et al. (15)	Single center, prospective, non-randomized	779	255	AT, AVNRT, WPW, AFL	NA	NA	EnSite NavX, CARTO	NA	440/332	NA	52 ± 19	NA	NA
Giaccardi et al. (16)	Multicenter, retrospective, non-randomized	442	297	AT, AVNRT, AVRT, AFL	3	NO	EnSite Velocity	NA	104/338	NA	59 ± 19; 58 ± 19	NA	NA
Seizer et al. (17)	Single center, retrospective, non-randomized	184	91	AVNRT, WPW, AT, AFL	NA	NA	EnSite NavX and Velocity	NO	87/97	389 ± 217	52.1 ± 19.1; 36.0 ± 22.1	79.4 ± 20.4; 70.5 ± 21.3 (kg)	100%
See et al. (18)	Single center, prospective, non-randomized	200	79	AVNRT, AVRT	NA	NA	EnSite NavX, CARTO	NA	110/90	360	39.5 ± 16.3; 43.4 ± 17.9	NA	NA
Nagaraju et al. (19)	Single center, retrospective	83	63	AVNRT, AVRT	1	NO	CARTO	YES (only for transseptal puncture)	46/37	148 (ZF) 329 (F)	13.7; 16.9	NA	54%
Marini et al. (20)	Single center, retrospective, non-randomized	93	57	AVNRT, AVRT, AT, EPS, VT	NA	NA	EnSite NavX, CARTO	NA	57/26	720	NA	65 (55–70); 57 (54–60) (kg)	NA
Swissa et al. (21)	Single center, prospective, non-randomized	139	64	AVNRT	2	NA	EnSite NavX	NA	68/71	360	12.8 ± 3.5 (4.3–17.8); 12.9 ± 3.8 (5–17.9)	19.5 ± 1.9 (15.7–22.1); 20.1 ± 4.1 (12.4–30.5) (BMI)	NA
Walsh et al. (22)	Single center, retrospective, non-randomized	92	50	AT, AVNRT, AVRT, EPS	1	NO	EnSite Precision	YES (only for transseptal puncture)	55/37	147	56 (36-69); 66 (49–74)	NA	94%
Tseng et al. (23)	Single center, retrospective, non-randomized	109	41	AVNRT, AT	NA	NA	EnSite Precision	NA	56/47	321	12.5; 12	53.1; 46.1 (kg)	100%
Pires et al. (24)	Single center, prospective, randomized	23	12	SVT, AFL, RVOT, AT	NA	NA	EnSite NavX	NA	9/14	NA	48.5 ± 1.6; 46.3 ± 16.6	NA	100%
Dengke et al. (25)	Single center, retrospective, non-randomized	227	112	left-AVRT	NA	NO	EnSite NavX	NA	135/92	90	50.2 ± 18.9 55.6 ± 17.9	NA	NA
Ceresnak et al. (26)	Multicenter, retrospective, non-randomized	651	366	AVRT	NA	NA	EnSite NavX, CARTO	NO	378/273	42 ± 36	13.0 ± 4.0	54.3 ± 23.3 (kg)	NA
Cauti et al. (29)	Single center	20	10	AVNRT, AT, AVRT, AFL	4	NA	Rhythmia	NA	NA	180	58 ± 12	NA	80%
Chen et al. (28)	Multicenter, prospective, non-randomized	3,060	1,020	AVNRT, AVRT	NA	NA	EnSite NavX	YES	1,367/1,693	291 ± 120	45.3 ± 5.4	63.8 ± 11.7 (kg)	99.3%
Fadhle et al. (27)	Single center, prospective, non-randomized	300	200	AVNRT, AVRT	4	NO	EnSite NavX, CARTO	NA	118/282	360	45.3 ± 15.4	63.8 ± 11.7 (kg)	99.5%
Di Cori et al. (30)	Single center, retrospective, non-randomized	206	93	EPS, AVNRT, AVRT, AT, AFL	NA	NA	CARTO, EnSite NavX/Velocity/Precision	NA	107/99	360	53 ± 19	26 ± 3.4; 25 ± 3.5	58%
Bergonti et al. (31)	Single center, retrospective, non-randomized	618	206	AVNRT, AVRT	NA	NA	EnSite NavX, CARTO	NA	247/371	1,584	38 ± 15	NA	67.5%

*Abl, ablation; AFL, atrial flutter; AP, accessory pathway; AT, atrial tachycardia; AVNRT, atrioventricular nodal reentrant tachycardia; AVRT, atrioventricular reentrant tachycardia; BMI, body mass index; EPS, electrophysiology study; NA, not available; RVOT, right ventricular outflow tract-ventricular tachycardia; WPW, Wolff–Parkinson–White syndrome*.

### Efficacy and Safety Outcome Events

**Figure 2:**
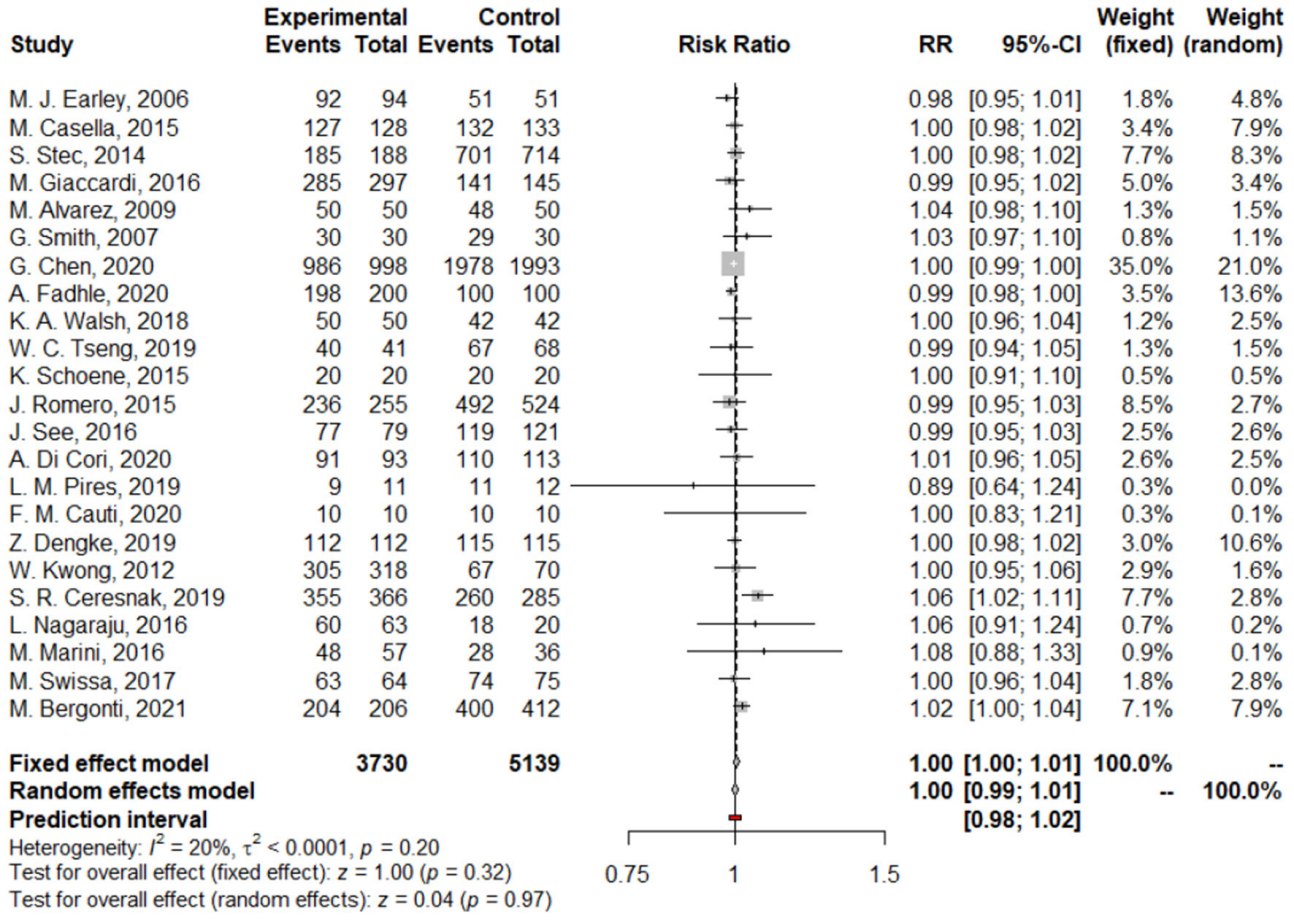
Forest plots of acute success rate.

**Figure 3:**
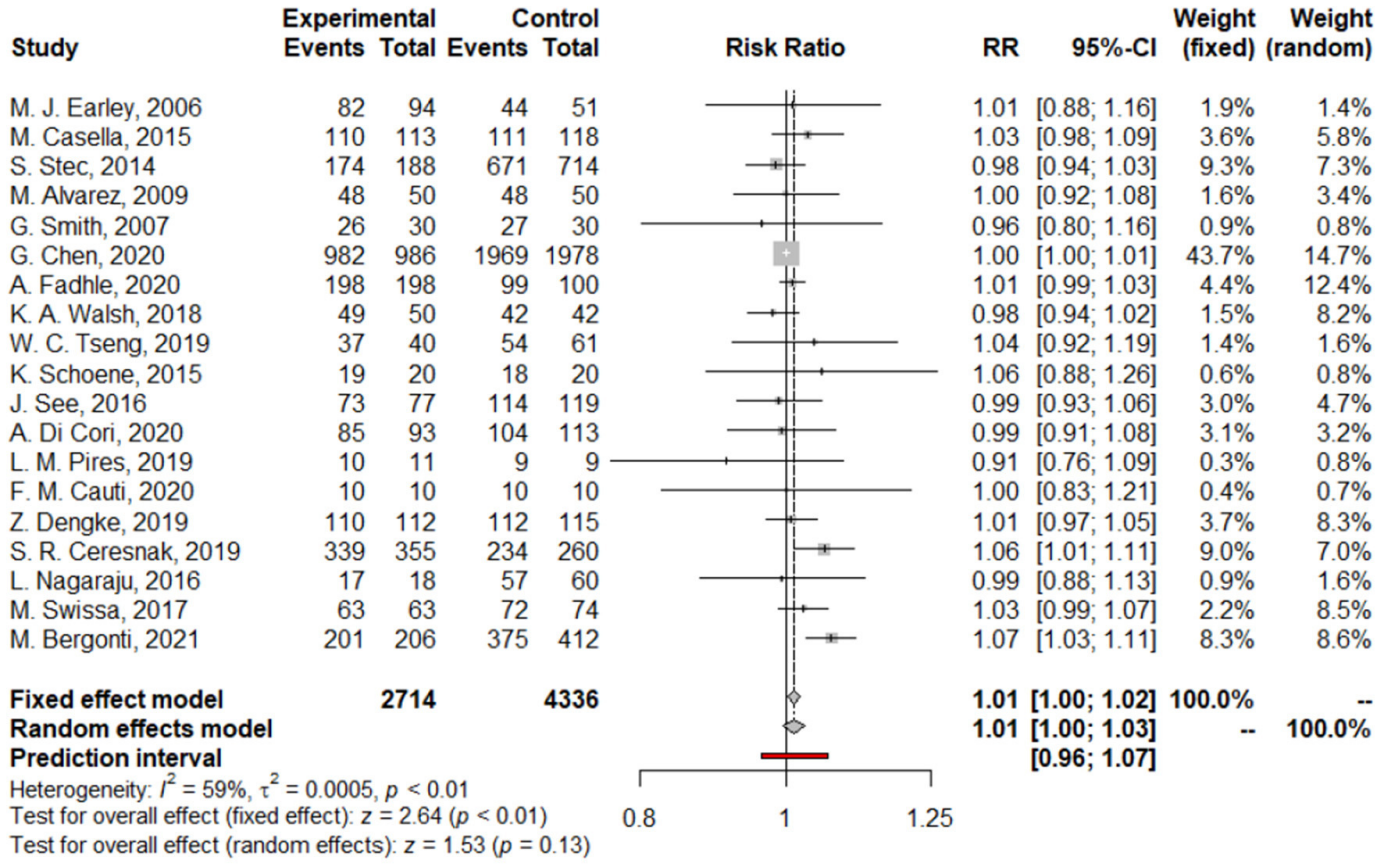
Forest plots of long-term success rate.

**Figure 4:**
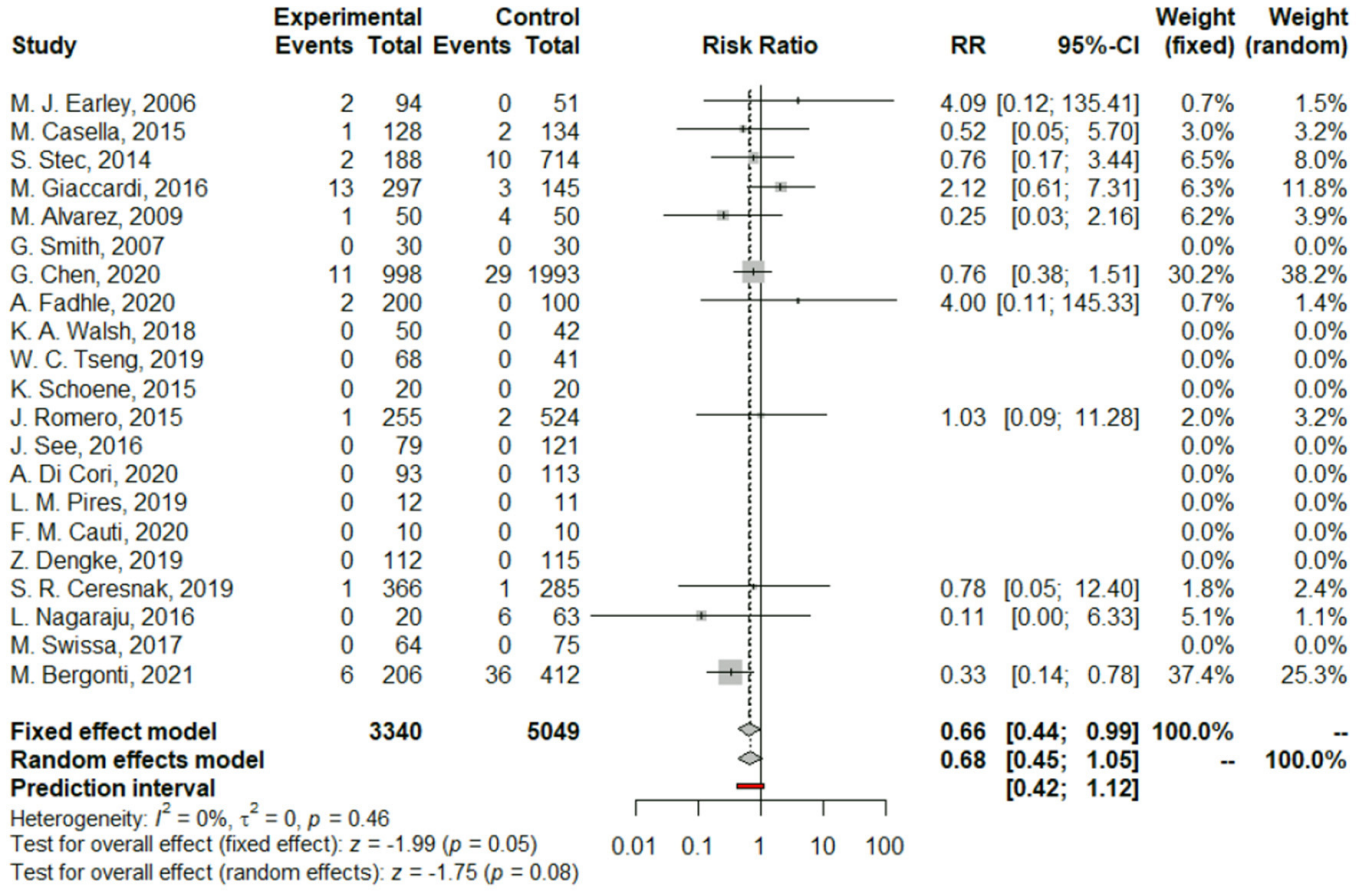
Forest plots of complications.

We found no difference between Z/MF and conventional ablation procedures in acute success rate (97.4 vs. 97.55%; RR: 1.00, 95% CI: 0.99–1.01, *p* = 0.97; Figure 2) and long-term success rate (97.02 vs. 96.17%; RR: 1.01, 95% CI: 1.00–1.03, *p* = 0.13; Figure 3). Complication rate was 1.59% in the entire study population and did not differ among the groups (RR: 0.68, 95% CI: 0.45–1.05, *p* = 0.08; Figure 4).

We performed a leave-one-out analysis, which showed similarly no difference between the groups for acute success rate (Supplementary Figure S1).

### Procedural Parameters

Compared to the conventional method, the Z/MF approach significantly reduced fluoroscopic time [MD: −1.58 min (95% CI, −2.21 to −0.96 min; *p* < 0.01)], fluoroscopy dose [MD: −10.95 mGy (95% CI: −18.43 to −3.46 mGy)], and radiation exposure [DAP; MD: −52.39 cGy/cm^2^ (95% CI: −65.38 to −39.40 cGy/cm^2^)]. Ablation time was shorter with the Z/MF method [MD: −25.23 s (95% CI: −42.04 to −8.43 s; *p* < 0.01)], whereas no difference could be detected between the two groups in terms of the number of ablation applications [MD: 0.13 (95% CI: −0.86 to 1.11; *p* = 0.80)] and procedure time [MD: 3.06 min (95% CI: −0.97 to 7.08 min; *p* = 0.14)] (Table 2).

**Table 2:** Summary of outcomes of secondary endpoints.

**Outcome**	**Number of**	**Number of**	**Mean difference**	**Test for**	**Heterogeneity**
	**studies**	**patients**	**(95% CI)**	**overall effect**	
Ablation time	7	4,750	−25.23 s (−42.04; −8.43)	*p* < 0.01	*I*^2^ = 40%; *p* < 0.12
Ablation application number	8	4,098	0.13 min (−0.86; 1.11)	*p* = 0.80	*I*^2^ = 71%; *p* < 0.01
Fluoroscopy time	17	7,326	−1.58 min (−2.21; −0.96)	*p* < 0.01	*I*^2^ = 98%; *p* < 0.01
Fluoroscopy dose	5	1,154	−10.95 mGy (−18.43; −3.46)	*p* < 0.01	*I*^2^ = 97%; *p* < 0.01
DAP	5	1,651	−52.39 cGy/cm^2^ (−65.38; −39.40)	*p* < 0.01	*I*^2^ = 100%; *p* = 0
Procedure time	15	7,290	3.06 min (−0.97; 7.08)	*p* = 0.14	*I*^2^ = 91%; *p* < 0.01

*DAP, dose area product*.

Funnel plot analyses and Egger's regression test showed no sign of possible publication bias (Supplementary Figure S1).

## Discussion

Our meta-analysis of 24 studies with 9,074 patients who underwent EP intervention due to paroxysmal SVT demonstrates that Z/MF ablation can significantly reduce radiation exposure, fluoroscopy, and ablation time. Compared to the fluoroscopy-guided ablation, the use of the Z/MF method proved to have no impact on the procedure time, the risk of complications, the acute or long-term success rate, and the number of ablation applications.

Medical exposure is the highest manmade source of radiation, representing a mean effective dose of 1–3 mSv per person per year (32). Radiation increases the life-time risk of cataract, dermatitis, and cancer *via* stochastic and deterministic effects (33–35).

Over the past decades, the reduction of the ionizing radiation during EP procedures has become a center of interest. Intraoperative mapping systems enable the visualization of the real-time anatomy of vessels and chambers of the heart, and the movement of the catheters. Owing to the fact that the use of EAM systems does not affect the procedure safety and efficacy, their use has become the most common method to achieve zero- or limited fluoroscopic guidance during cardiac ablation (36).

Preferring ZF guidance to traditional fluoroscopic approach is extremely important in high-risk populations, particularly in pregnant women and children (37, 38). According to the latest guideline of the European Society of Cardiology (ESC), fluoroless catheter ablation should be performed in pregnant women with drug-refractory or poorly tolerated SVT (1).

A previous meta-analysis of 2016, with the inclusion of 2,261 patients, compared the Z/MF and fluoroscopic approaches during ablation of cardiac arrhythmias (5). In correspondence with our recent findings, this meta-analysis also showed a significant reduction of fluoroscopy and ablation time, whereas the procedure time, ablation time, complications, and acute and long-term success rates were similar between the two groups. However, we had the opportunity to involve significantly more patients, which strengthens the generalizability of these results to SVTs.

### Procedural Parameters

Theoretically, the use of EAM systems may reduce the procedure length due to 3D visualization and allows easier return to a desired place with the catheters. On the other hand, the creation of an EAM systems requires several minutes contrary to the conventional, fluoroscopy-guided method. In our analysis (in which atrial fibrillation ablation procedures were not included), we found no difference in terms of the procedure time between the groups; however, a significant heterogeneity was detected among the 18 studies in this regard: some studies found longer procedure time (9–12, 15, 16, 22, 28, 30), whereas other trials showed significantly reduced procedure time, with the Z/MF method (13, 17, 18, 21, 23, 25). This may be attributed to the heterogeneity of the performed ablation procedures, including AT, AVNRT, AVRT, and AFL, and the different methods of performance of these interventions among different centers. This fact may also explain that ablation time was found shorter with the use of EAM systems, despite the fact that similar amount of ablation applications occurred in the groups. We found much more favorable results in terms of procedural parameters in the Z/MF group; these findings were consistent between the studies included.

### Acute Success

Acute success rate was above 97% in the entire study population. We found no difference between the groups. Among 23 trials reporting acute success rate, comparing the fluoroscopy-only and the Z/MF-only approaches, Ceresnak et al. (26) demonstrated a significant difference analyzing children with the Wolff–Parkinson–White syndrome (26). In this multicenter retrospective trial, the use of EAM systems improved the acute success rate; however, the rate of the procedures utilizing cryoenergy was higher in the fluoroscopy-only group, and cryoablation was associated with decreased success rate on multivariable analysis (26).

### Complications

Complication rate was low (1.59%) and did not differ significantly among the groups. No significant heterogeneity was detected among 21 studies reporting complications. Interestingly, a retrospective observational trial by Bergonti et al. (31) found higher rate of complications in the conventional arm compared to the Z/MF approach (8.73 vs. 2.91%). This difference mainly comprised late complications (i.e., advance AV block and need for pacemaker implantation). According to the authors, these results may be explained by the fact that, with EAM systems, the proximity of the His bundle area can be safely monitored all along the procedure.

### Long-Term Success

Eighteen trials included in our analysis reported on long-term success results, and only two of them, including patients with AVNRT and AVRT, found difference between conventional and Z/MF ablation procedures, namely the Z/MF approach, which was associated with a lower recurrence rate (26, 31). Surprisingly, Bergonti et al. (31) reported 8.98% recurrence rate during the 52-month follow-up in the conventional arm (31), which is much higher than the literature data (1). This difference may be explained by the fact that recurrence was defined as “experience recurrence of arrhythmias” even without electrocardiography (ECG) documentation. Nevertheless, our analysis showed no difference in long-term success rate between Z/MF and conventional, fluoroscopy-guided methods.

To analyze the potential impact of this trial to the results of the meta-analysis and its heterogeneity, in-depth analyses were carried out (Supplementary Figures S3–S5). Based on these, the study of Bergonti et al. (31) was identified to be among the five studies being the potential source of the data heterogeneity; however, the impact on the results was negligible. Moreover, in leave-one-out analyses, the omission of this study did not impact our results (Supplementary Figure S2).

To acquire the skills to be able to properly apply the Z/MF-guiding technique, the operators have to complete a learning curve, which comprises 20 procedures in the case of SVTs (39). Besides the operators' experience, the type of the arrhythmia and the center volume may also have an effect on the success of zero-fluoroscopy strategy (40). The most challenging part of the total fluoroless ablation procedure is the transseptal puncture; however, this step can be guided by intracardiac echocardiography (ICE). In addition, the combination of EAM systems and ICE provides an even more accurate approach compared to the standard fluoroscopy views (41).

The use of EAM systems may increase EP procedure costs; however, Casella el al. (13) found that the additional cost of the Z/MF method is approximately equal to the extra costs associated with the increased cancer treatment and the reduction in the quality of life associated with conventional fluoroscopy-guided techniques (13). We believe that this higher cost should not be a barrier to improve the safety of both patients and medical staff in EP procedures.

## Limitation

Some aspects of our study should also be discussed as they may serve as possible limitations. First, only three randomized studies were included, and the majority of data originate from observational studies. This may introduce potential biases and/or effects of unmeasured confounders. Important differences may also exist in patient demographics that might affect outcomes and are not accounted for in this analysis (e.g., body mass index, ethnicity, or gender). Second, a high degree of heterogeneity was observed (>50%) between the different study populations. The use of a random-effects model can help mitigate the potential effect of heterogeneity, and the high level of significance supports the validity of the results. Third, outcomes were not reported by all of the included studies, limiting further analysis of potential mechanisms. Finally, data regarding the operators performing the procedures and especially data on operators' previous experience with the Z/MF approach were also insufficient despite the probable impact of a learning curve effect.

## Conclusion

In conclusion, our meta-analysis including 9,074 patients demonstrated that the Z/MF approach for the treatment of SVT is a feasible method that reduces fluoroscopy time, radiation exposure, and ablation duration but does not compromise the acute and long-term success or complication rates.

## Data Availability Statement

The original contributions presented in the study are included in the article/Supplementary Material, further inquiries can be directed to the corresponding author.

## Author Contributions

PK, TS, and DD contributed to conception and design of the study. AK performed the statistical analysis. DD, PK, MV, and AK wrote sections of the manuscript. Tables and figures were designed by PK, DD, and KJ. All authors contributed to manuscript revision, read, and approved the submitted version.

## Conflict of Interest

MV reports consulting fees and/or nonfinancial support from Biotronik, Medtronic, and Pfizer, outside the submitted work. The remaining authors declare that the research was conducted in the absence of any commercial or financial relationships that could be construed as a potential conflict of interest.

## Publisher's Note

All claims expressed in this article are solely those of the authors and do not necessarily represent those of their affiliated organizations, or those of the publisher, the editors and the reviewers. Any product that may be evaluated in this article, or claim that may be made by its manufacturer, is not guaranteed or endorsed by the publisher.
