# Supplementary material for: Zero and Minimal Fluoroscopic Approaches During Ablation of Supraventricular Tachycardias: A Systematic Review and Meta-Analysis
Source: Front Cardiovasc Med. 2022 Apr 11;9:856145. doi: 10.3389/fcvm.2022.856145 (PMC9037593; doi:10.3389/fcvm.2022.856145)
Supplement: Supplementary file 1 [file Data_Sheet_1.DOCX]

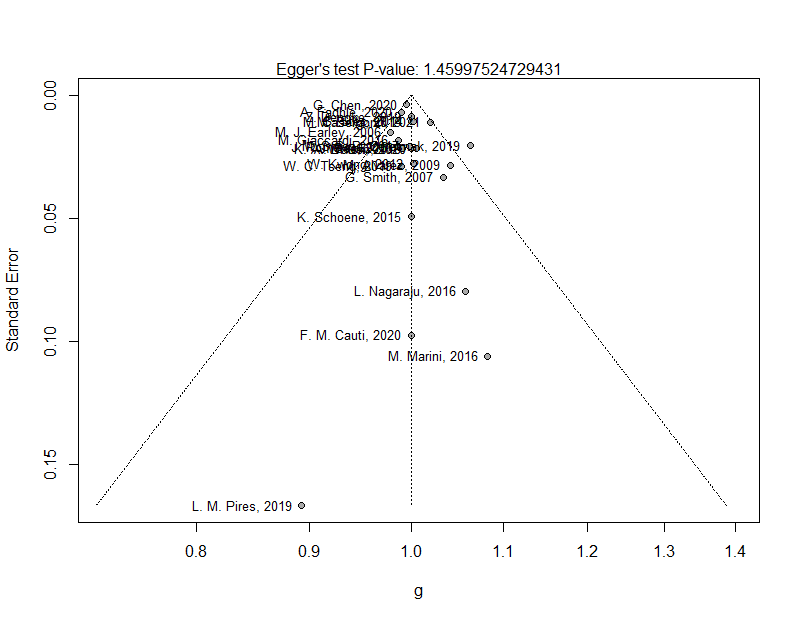


**Supplementary Figure 1. Funnel plot analysis of publication bias**

The funnel plot shows the effect estimates from individual studies plotted against each study's precision. Neither visual estimation nor Egger’s regression test suggested important publication bias.


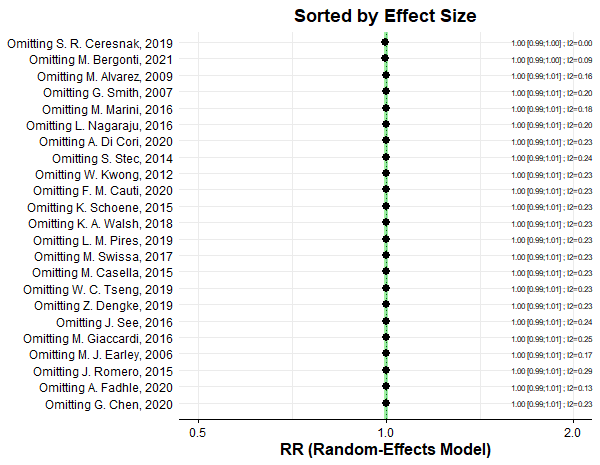


**Supplementary Figure 2. Forest plot representation of the Leave-one-out analysis of the acute success in trials with low-fluoroscopy strategy.** Leave one out analysis showed consistent results with omission any of the trials.

## Analysis of between-study heterogeneity

The between-study heterogeneity variance was estimated low (τ^2^<0.01 with an I^2^ = 5.9% [0.0%; 38.4%] and H = 1.03 [1.00; 1.27]). Similarly, Cochrane’s test p value was high (p=0.38). The prediction interval ranged from g=[0.9943; 1.0143], indicating low risk of for future studies showing different efficacy for low-fluoroscopy procedures.

In Graphic Display of Heterogeneity (GOSH) plot we fitted the same meta-analysis model to all possible subsets of our included studies. The GOSH plot reflected a roughly symmetric, distribution, however, the scattering of the effect estimate hinted for more positive result in the higher heterogeneity subgroups. (Supplementary Figure 3.)


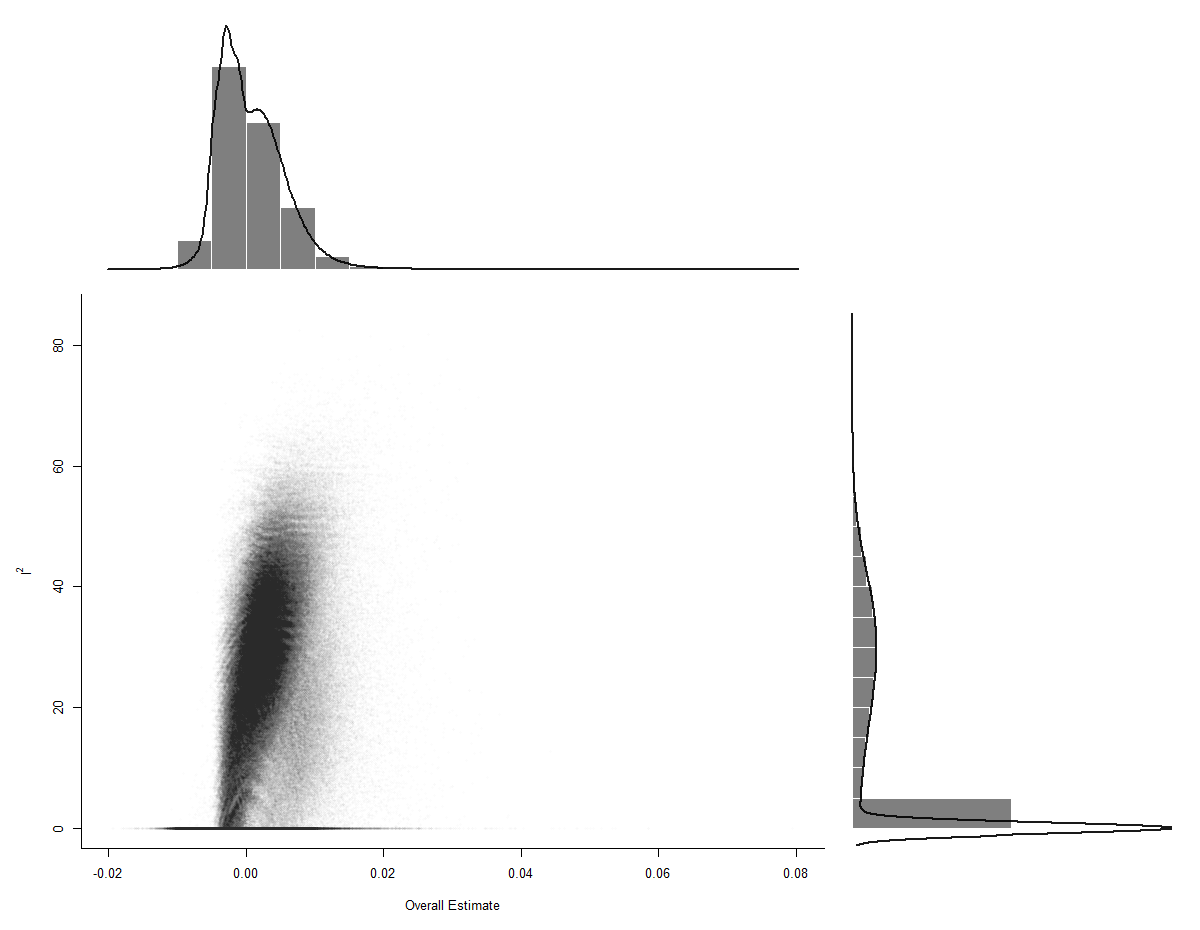


**Supplementary Figure 3. The ‘Graphic Display of Heterogenety’ plot displays the pooled effect size on the x-axis and the between-study heterogeneity on the y-axis in randomly selected 10^6^ sample of studies.**

Cluster identification algorithms including k-means algorithm, gaussian mixture models and as well as ‘density reachability and connectivity clustering’ or DBSCAN similarly identified S. R. Ceresnak, 2019 et al. (Study 19) to be an influential case. Additionally, M. J. Earley et al, 2006 (Study 1), G. Chen et al. 2020 (Study 7), A. Fadhle et al, 2020 (Study 8), and M. Bergonti et al, 2021 (Study 23) showed being over-represented in clusters with high heterogeneity. This indicates that these studies, alone or in combination, may be responsible for the higher heterogeneity. (Supplementary Figure 4)


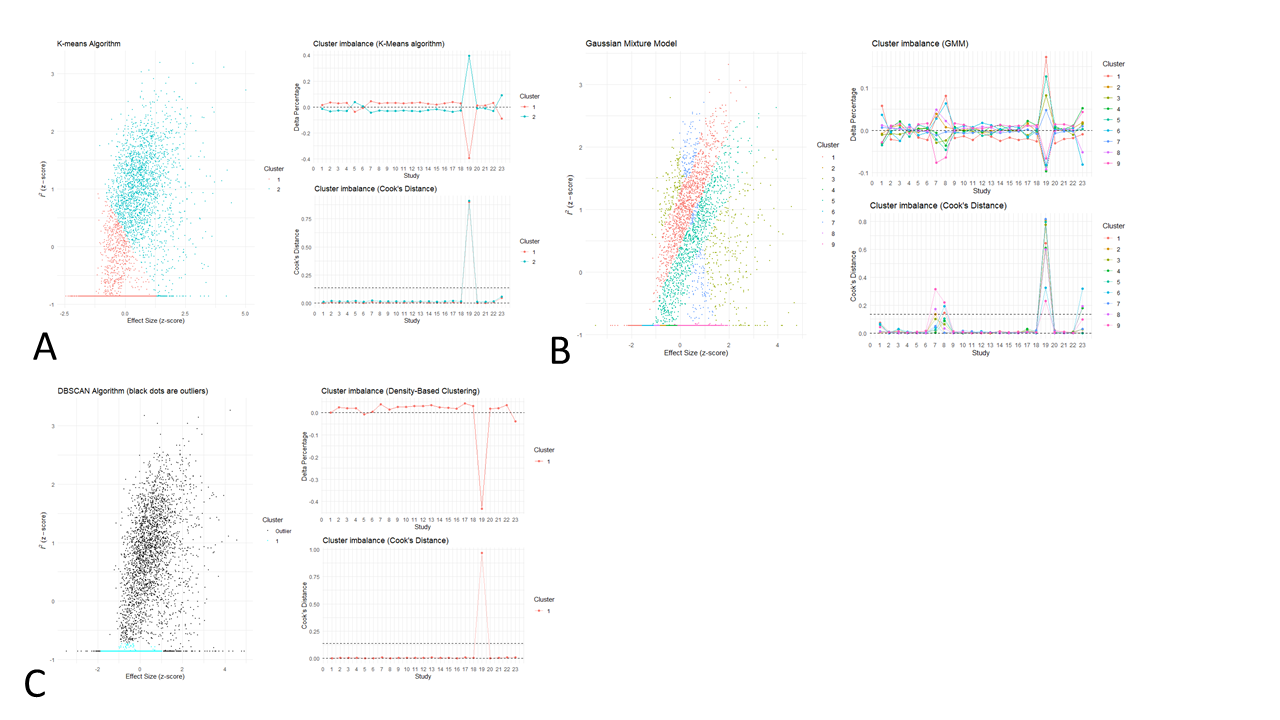


**Supplementary Figure 4. Cluster identification algorithm analysis of the ‘Graphic Display of Heterogeneity’ outputs**

Plotting the GOSH plots again marking subsets with these studies included, showed that no individual study is responsible for the clustering. (Supplementary Figure 5 panel C-D)


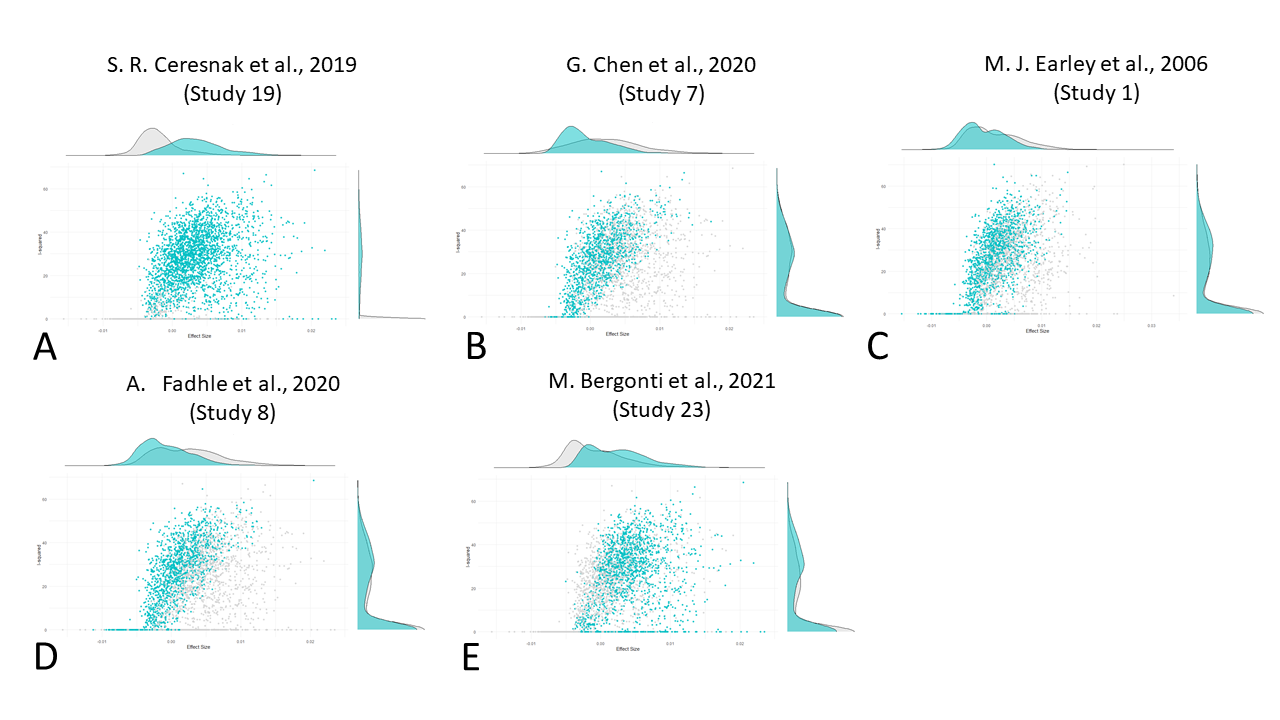


**Supplementary Figure 5.** Cluster identification algorithm analysis of the ‘Graphic Display of Heterogenety’ with outputs plotted with marking subsets including the influential studies marked in the gaussian mixture models analysis

Outlier analysis pinpointed also the study of S. R. Ceresnak, 2019. Removal of this trial resulted in reduction of heterogeneity (I2=0.0%, H=1.0, and τ^2^=0), however, did not impacted the overall effect estimate RR: 0.9973 [0.9921; 1.0024] p=0.51, and prediction interval g= [0.9918; 1.0027]. (Supplementary Figure 5 and 6.)


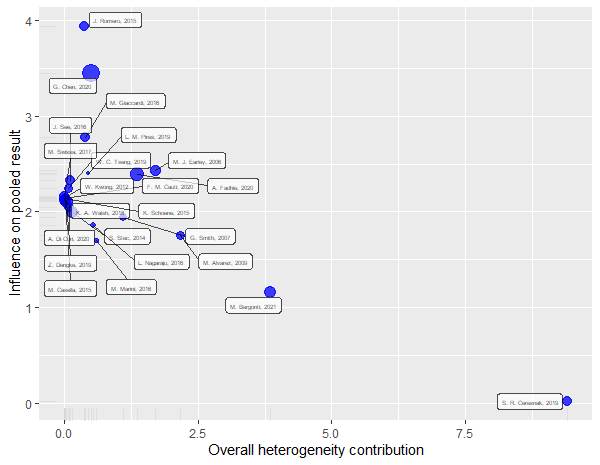


**Supplementary Figure 6. Baujat plot of trials showing the contribution of individual trials to the pooled results and the heterogeneity**
